# Neurotoxic Potential of Deoxynivalenol in Murine Brain Cell Lines and Primary Hippocampal Cultures

**DOI:** 10.3390/toxins14010048

**Published:** 2022-01-10

**Authors:** Christiane Kruse Fæste, Anita Solhaug, Marion Gaborit, Florian Pierre, Dominique Massotte

**Affiliations:** 1 Toxinology Research Group, Norwegian Veterinary Institute, 1433 Ås, Norway; anita.solhaug@vetinst.no; 2 Centre de la Recherche Nationale Scientifique, Institut des Neurosciences Cellulaires et Intégratives, University of Strasbourg, 67000 Strasbourg, France; gaborit@inci-cnrs.unistra.fr (M.G.); f.pierre@ibmc-cnrs.unistra.fr (F.P.)

**Keywords:** astrocytes, deoxynivalenol, microglia, mouse, mycotoxin, primary hippocampal cultures

## Abstract

Chronic exposure to the mycotoxin deoxynivalenol (DON) from grain-based food and feed affects human and animal health. Known consequences include entereopathogenic and immunotoxic defects; however, the neurotoxic potential of DON has only come into focus more recently due to the observation of behavioural disorders in exposed farm animals. DON can cross the blood-brain barrier and interfere with the homeostasis/functioning of the nervous system, but the underlying mechanisms of action remain elusive. Here, we have investigated the impact of DON on mouse astrocyte and microglia cell lines, as well as on primary hippocampal cultures by analysing different toxicological endpoints. We found that DON has an impact on the viability of both glial cell types, as shown by a significant decrease of metabolic activity, and a notable cytotoxic effect, which was stronger in the microglia. In astrocytes, DON caused a G1 phase arrest in the cell cycle and a decrease of cyclic-adenosine monophosphate (cAMP) levels. The pro-inflammatory cytokine tumour necrosis factor (TNF)-α was secreted in the microglia in response to DON exposure. Furthermore, the intermediate filaments of the astrocytic cytoskeleton were disturbed in primary hippocampal cultures, and the dendrite lengths of neurons were shortened. The combined results indicated DON’s considerable potential to interfere with the brain cell physiology, which helps explain the observed in vivo neurotoxicological effects.

## 1. Introduction

**Figure 1:**
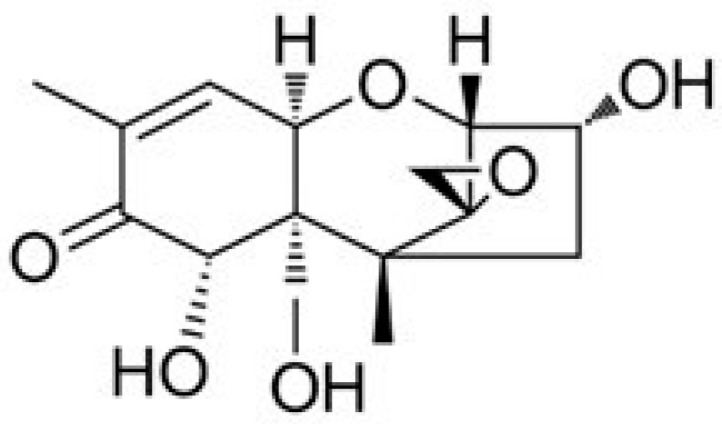
Molecular structure of deoxynivalenol (DON).

The mycotoxin deoxynivalenol (DON) (Figure 1) occurs with high a prevalence in grain-based products worldwide [1]. Consequently, the maximum allowed levels in food and feed have been implemented by national and international authorities, based on toxicity studies and risk assessments, as performed by the European Food Safety Authority (EFSA) [2,3,4]. Compliance with the regulations generally prevents incidences of acute DON intoxication, but low-level chronic exposure in humans and animals is still common. The monitoring of urinary biomarkers of DON allows evaluating the amounts of ingested DON [5]. Surveys performed in different countries have shown that the mean chronic dietary exposure, especially in children, often exceeds the established tolerable daily intake (TDI) of 1 µg/kg body weight (bw) for the sum of DON and its derivatives [6,7].

Several in vivo studies have shown that chronic low-level DON exposure can lead to entereopathogenic, immunotoxic, and neurotoxic effects, resulting in negative changes in gut health, immunological resilience, and behaviours [8,9,10]. DON-induced neurotoxicity has come into focus only recently, triggered by observed deviant behaviour traits, such as aggressiveness, anxiety, and increased locomotor activity in mice, rats, pigs, poultry, and mealworm beetle larvae after chronic exposure to feedborne DON at concentrations ranging from 0.1 to 25 mg/kg [10,11,12,13,14]. DON can cross the blood-brain barrier and directly modulate brain activity [15,16]. Known consequences of chronic low-dose DON exposure include the disturbance of catecholamine neurotransmitter levels such as dopamine, norepinephrine, and serotonin in the hypothalamus and cortex of different animal species [17,18,19]. Furthermore, pro-inflammatory cytokines (interleukin (IL)-1β, IL-6, tumour necrosis factor (TNF)-α), and the transcription factor c-Fos were increased in specific brain regions, including the *nucleus accumbens*, paraventricular nucleus of the thalamus, and dorsal *tenia tecta* after repeated exposure to low DON concentrations [9,10,12,20,21,22,23]. Feeding piglets with >1.3 mg DON/kg feed for 60 days disrupted the cerebral calcium homeostasis, increased lipid oxidation, and decreased the activities of the anti-oxidative enzymes glutathione peroxidase and superoxide dismutase [23]. In rats treated with >0.2 mg DON/kg bw for 42 days, a reduction of the gliocytes and neurons was observed in the myenteric plexus of the jejunum [24].

Several in vitro assays have been performed to determine the underlying molecular mode of actions for the detected physiological changes caused by DON. The mycotoxin is water-soluble and uncharged at physiological pH. However, studies in a cell line, derived from colon cells (Caco-2) have shown that DON can pass through cell membranes and reach the cytosol as a substrate of ATP-binding cassette transporters (ABC transporters) [25]. Intracellularly, one primary action of DON is inhibition of the protein synthesis by binding to the 60S ribosomal subunit and decreasing peptidyl transferase capacities, leading to activation of mitogen-activated protein kinase (MAPK) and subsequent apoptosis [26]. When DON-triggered effects on transcription and translation reactions were investigated in mouse macrophages by phosphoproteomics, changes in the phosphorylation patterns of almost 200 proteins were observed, including those involved in proliferation, cell cycle, differentiation, and cytoskeleton organisation [27]. Apoptosis through the mitochondrial pathway was prompted by the activation of caspase-3 leading to DNA fragmentation in PC12 cells of rat adrenal medulla [28]. Furthermore, DON was found to cause oxidative stress and damage in chicken embryo fibroblast DF-1 cells [29].

The consequences of exposing macrophages to low DON levels have been investigated in several in vitro studies, showing an increased production and secretion of inflammatory cytokines such as IL-1β, IL-6, and TNF-α, as well as changes in the surface expression of cell signalling and adhesion molecules as a main outcome [30,31,32,33,34]. Further reactions included the increase of hydrogen peroxide and nitric oxide (NO) levels in the presence of immunostimulants, e.g., lipopolysaccharide (LPS) or interferon [29,30,33,35]. In vivo, macrophage-driven inflammation has been identified as an early reaction of chronic low-level DON exposure both in the gastrointestinal tract and brain of mice, rats, chicken, and pigs [9,12,18,20,36], demonstrating the predictive value of the cell toxicity studies [37].

Interestingly, most assays that elucidate consequences of DON exposure at the cellular level have been performed in intestinal, skin, kidney, or peripheral blood cells. Reports on effects in brain cells are scarce, although relevant DON concentrations can be measured in the brain already 5 min after oral uptake [36]. Furthermore, it has been shown that DON induced apoptosis in a hippocampal cell line from a pig, by triggering the inflammation-related MAPK signalling pathway [15]. Moreover, in rat primary cortical glial cells and immortalized human microglial C13NJ cells, low DON concentrations led to the production of reactive oxygen species from lipid oxidation and NO production [29,35]. Glial cells, including astrocytes and microglia, are essential for maintaining brain homeostasis and functionality as astrocytes provide nutrients and structural support, and microglia supply for the main immune defence in the central nervous system [38]. Disturbance of the physiological balance by DON could be one of the reasons behind the observed in vivo neurotoxicity [39].

In the present study, we have, therefore, focussed on investigating DON-induced effects on murine astrocytes and microglial cell lines, as well as on primary brain cells from young mice, while studying relevant endpoints of toxicity in several cytotoxic and functional tests.

## 2. Results and Discussion

### 2.1. Viability of Mouse Astrocytes and Microglia

The viability of both glial cell types in the presence of µmolar DON concentrations was assessed by measuring the metabolic activity using the Alamar blue assay and by determining the general cytotoxicity using the Celltox Green assay [40]. Results of the Alamar Blue assay reflect the general impact of a substance on the cells viability, independently of the actual mode of action, which e.g., could be connected to reduced cell proliferation, reduced metabolism, and/or increased cell death. On the other hand, the Celltox Green assay uses the influx of a non-toxic fluorescent dye into the cells with impaired membrane integrity as a measure for general cytotoxicity. 

**Figure 2:**
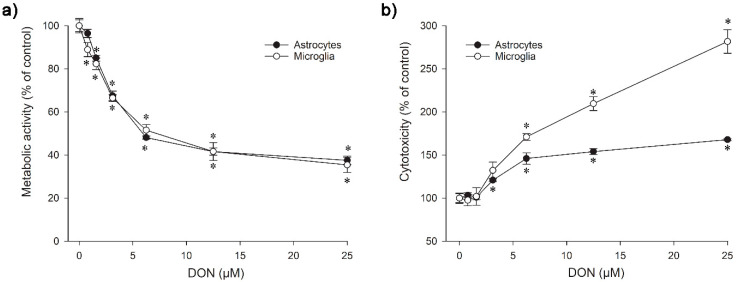
Effect of DON on the viability of astrocytes and microglia after 24 h of exposure; (**a**) Metabolic activity as measured in the Alamar Blue assay; (**b**) Cytotoxicity as determined in the Celltox Green assay. The data represent the mean ± SEMs of four independent experiments performed in triplicates. Significant differences (*p* < 0.05) as compared to the control are indicated with asterisks (*).

Both the murine astrocytes and microglia showed a significant decrease of metabolic activity already after exposure to 0.8 µM (IC_50_ 5.8 µM) and 1.6 µM DON (IC_50_ 7.2 µM) for 24 h, respectively (Figure 2a). The microglia were notably more sensitive in the cytotoxicity assay than the astrocytes, with significant differences to the untreated control at 3.1 µM DON for microglia and at 6.3 µM DON for astrocytes after exposure for 24 h (Figure 2b). A distinct dissimilarity between the nervous cell types was observable from the further progression of the concentration-cytotoxicity curve, leading to a wide sensitivity gap at 25 µM.

Considering the results of the Alamar Blue and the Celltox Green tests together, it appeared that the viability decrease of the microglia resulted largely from DON-induced cytotoxicity at concentrations above 3.1 µM, whereas the rather steep drop in viability observed for the lower DON levels was probably caused by reduced metabolic activity. In the astrocytes, the cytotoxic effects were much less pronounced, as similarly seen at higher DON concentrations, so that they alone are not sufficient to explain the detected viability loss. 

A decrease of cell viability connected to a reduced metabolic activity from exposure to DON has previously been determined in human colorectal adenocarcinoma (Caco-2) cells at 10 µM [41], in dog kidney (MDCKII) cells at 4 µM [25], in pig pulmonary alveolar macrophages (PAM) at 1 µM [30], in human (Jurkat T cells or acute monocyte leukaemia cells (THP-1)), and mouse (RAW 264.7) macrophages at 0.8 µM [31,32,42]. The differences in the measured sensitivities may reflect that different tests, e.g., Alamar Blue or Thiazolyl blue tetrazolium bromide (MTT), were used to measure the effect of DON on the cell metabolic activity, but could also originate from an increased vulnerability in cells with high metabolic activity. The astrocytes have a considerable high metabolism, due to their role in nutrient provision, and thus damage repair in the brain might significantly be affected by DON (Figure 2a), while the cells were more resistant to DON-initiated processes causing membrane damage (Figure 2b). As previously shown in human epithelium cells of the small intestine (HT-29-D4), DON can disturb the nutrient absorption by interacting with transmembrane transporters, such as the D-glucose/D-galactose sodium-dependent transporter (SGLT-1), D-fructose transporter (GLUT-5), or the D-glucose transporter 2 (GLUT-2) [43]. A similar interference could be supposed to impair transport mechanisms across the astrocyte cell membrane, namely via the D-glucose transporter GLUT-1 [44]. DON at concentrations of 1 µM or higher has been shown to prevent astrocytes from reabsorbing the neurotransmitter glutamate [35].

Our results showing a disparity in DON sensitivity between murine astrocytes and microglia in the cytotoxicity assay agreed well with previous reports. When primary rat microglia and astrocytes were exposed to DON for 48 h, IC_50_ of 0.6 µM versus 31 µM were measured, and the exposure of immortalised human microglial (C13NJ) and astrocytic (STTG-1) cells led to IC_50_ 4.1 µM versus 50 µM [35]. A conclusive explanation for the increased DON sensitivity of the microglia has not been offered yet, but it could be connected to the toxin’s ribotoxic activity, since the same was found for other agents with a congruous mode of action [33,35]. Glial reactivity is regarded as the key indicator for brain inflammation and as an early biomarker of neurotoxicity [45,46]. Tests involving other monocytes/macrophages demonstrated cytotoxicity at similar DON concentrations, resulting in significant effects at 2 µM in human monocyte leukaemia cells (THP-1), measured by the same Celltox Green assay as used in our study [31]. Alternative test methods for cytotoxicity led to the determination of IC_20_ at 0.4 µM DON in primary porcine macrophages [30] and a significance at 0.2 µM in mouse macrophages (RAW 264.7) [47]. A plausible explanation for the detected differences in DON-related cytotoxicity between astrocytes and microglia could be the much higher proliferation rate of the astrocytes, which makes them more robust due to higher turnover than the slow-proliferating microglia.

### 2.2. Cell Cycle Analysis in Astrocytes

Looking for possible causes for the somewhat low DON-induced cytotoxicity in the murine astrocytes, we next studied potential effects disturbing the normal sequence of the cell cycle. Healthy, dividing cells synthesise RNA and proteins during the post-mitotic G1 phase, duplicate the DNA in the S phase, increase the production of RNA and proteins in the G2 phase, and undergo mitosis in the M phase. To avoid the generation of cells with damaged or abnormal DNA, the cell cycle progression in mammalian cells is under strict control by numerous factors, including mediator, effector, and regulator proteins, e.g., phosphokinases, transcription factors, and histones [48]. The arrest or delay of the cell cycle provides time for DNA repair or the initiation of apoptosis to prevent the proliferation of malign copies. Accurate transition from the G1 phase to the S phase is thus a crucial point in the cell cycle.

**Figure 3:**
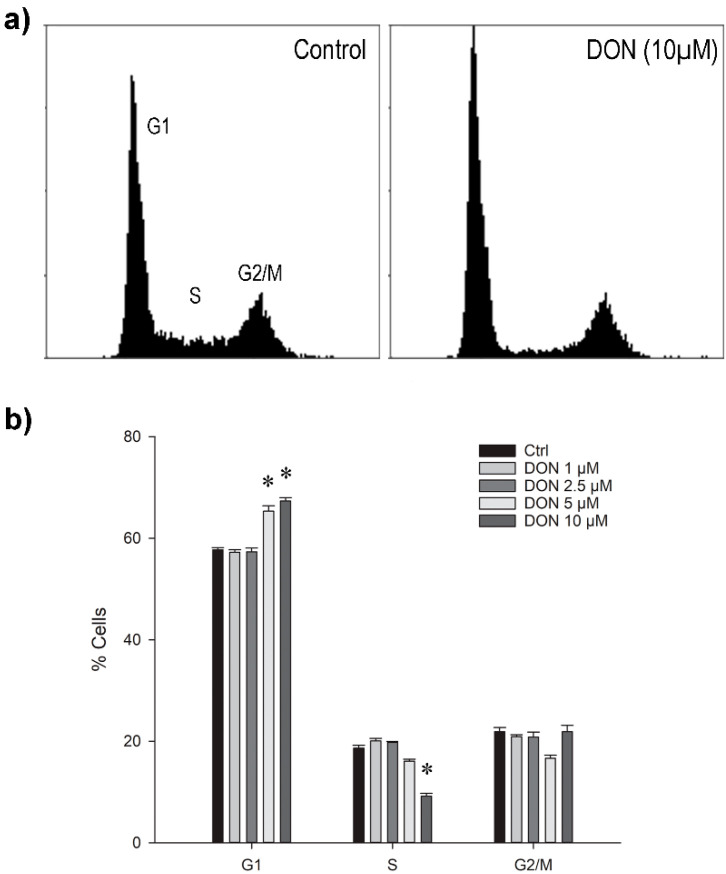
(**a**) Typical distribution of murine astrocytes after 24 h exposure to 0 or 10 µM DON in different phases of the cell cycle as measured by flow cytometry; (**b**) Ratios of the cell population in G1, S, and G2/M phases after exposure to different DON concentrations for 24 h. The data are representatives of two independent experiments and are expressed as mean ± SD of four replicate incubations. Significant differences (*p* < 0.05) as compared to the control are indicated with asterisks (*).

We observed a significant increase of cells in the G1 phase after 24 h exposure to 5 or 10 µM DON, accompanied by the respective decrease of cells in the S-phase (Figure 3a,b). The ratio of cells present in the G2/M phase was apparently not substantially changed. However, a general slow-down in proliferation was observed already at 1 µM DON (data not shown).

The DON-induced G1 arrest detected in the astrocytes in this study pointed at a substantial disturbance of on-going cellular processes; however, the underlying mechanism is unclear. Considering the ribotoxic activity of DON, interference with protein translation, activation of the MAPK pathway, and the subsequent disruption of regulatory functions that initiate the transition to the S phase could have caused the observed effect. Comparable to our findings, G1 arrest by low-level DON exposure for 24 h as well as the initiation of apoptosis has previously been described in human monocytes (THP-1) [31], mouse macrophages (J774A.1) [49], rat intestinal epithelia (IE-6) [50], and primary pig endometrial cells [51]. For human hepatocarcinoma cells (HepG2) [52] and chicken embryo fibroblasts (DF-1) [29], a significant increase in G1 was determined after exposure to 0.6 µM and 1.7 µM DON, respectively, whereas levels above 1.2 µM and 3 µM, respectively, led to a significant rise in the G2/M cell population and decline of cells in the S-phase accompanied by substantial cell death.

A similar change was found in intestinal pig cells (IPEC-J2), where exposure to 6.8 µM DON for 24 h resulted in the increase and decrease of cell numbers in the G1 and S phase, which was reversed and complemented with an increase of G2/M cells after incubation for 48 h and 72 h [53]. In contrast, incubation of mouse macrophages (RAW264.7) with 1 to 4 µM DON for 12 h blocked the cell cycle immediately in the G2/M phase [32], which was also observed in human intestinal epithelia (HCT-116) after exposure to 0.8 to 3.4 µM for 48 h [54]. Considering the different reports, the detected changes appeared to be influenced by a combination of DON concentrations, exposure periods, and sensitivity of the respective cell lines. Since the orderly organisation of the cell cycle depends on multifactorial coordination, the identification of the decisive part would be challenging [48]. Nevertheless, a recent transcriptomic study of Caco-2 cells that had been exposed to 0.5 µM DON for 24 h revealed changes in the expression of cell cycle-related genes as one of the top five affected pathways, creating the basis for more in-depth target analyses [55].

### 2.3. Cytokine Secretion in Microglia

**Figure 4:**
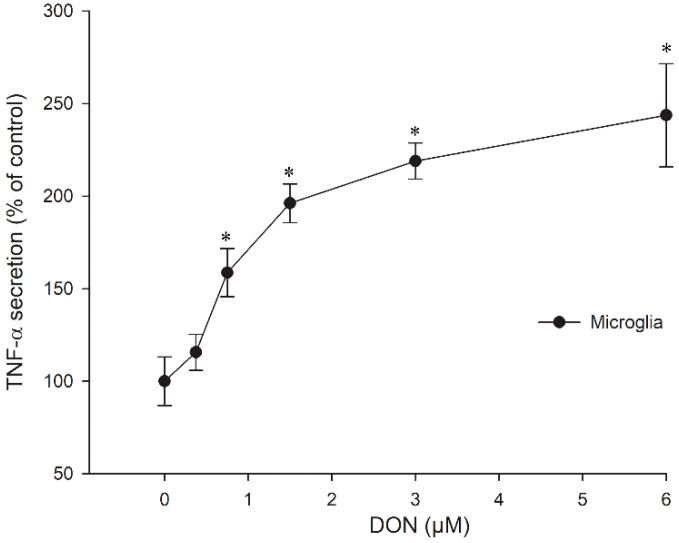
TNF-α secretion in microglial cells after 24 h exposure to different DON concentrations. The data represent the mean ± SEM of four independent experiments and are normalised to the control at 100 %. Significant differences (*p* < 0.05) as compared to the control are indicated with asterisks (*).

Based on the results of the cytotoxicity assay we investigated in a next step, whether DON-stimulated cytokine expression occurred in the murine astrocytes and microglia. While neither TNF-α nor IL-1β secretion were detectable in astrocytes after 24 h incubation (data not shown), dose-dependent TNF-α secretion was measurable in the microglia at non-cytotoxic DON concentrations **(**Figure 4). Interestingly, IL-1β secretion was not found, not even after pre-incubation with the immunostimulant LPS for 3 h (data not shown), which previously had led to increased synergistic release of IL-1β and TNF-α at low DON concentrations in a human acute monocyte leukaemia cell line (THP-1) [56].

Transcriptomic analysis of primary porcine macrophages incubated with 0.5 µM DON showed that the expression of TNF-α mRNA peaked after 3 h, and TNF-α protein in the cell supernatant was elevated until 48 h [30]. Similarly, IL-1β mRNA was highest after 3 h DON exposure, but IL-1β protein declined rapidly and was at 24 h almost at the same level as at 0 h. The additional use of LPS made no difference, which was congruent with our results determined in the murine microglia. The regulation of cytokine profiles in the microglia could be a means of adjusting the progression of neuroinflammation. Moreover, it has been suggested that astrocytes might be able to attenuate microglia activation [45].

### 2.4. cAMP Levels

Cyclic-adenosine monophosphate (cAMP) is part of intracellular signal transmission pathways, controlling the activity of protein kinase A as a co-factor and thus regulating the subsequent phosphorylation of multiple substrates that are relevant in stress response, cell proliferation, and development. Neurotoxic chemicals have been shown to affect cAMP levels in astrocytes in vivo [57] and to impair cAMP-dependent astrocytic differentiation in vitro [58]. Moreover, the stimulation of cAMP-prevented neuroinflammation in primary rat astrocytes by significantly reducing the LPS-inducible nitric oxide synthase (iNOS)/NO release [59]. 

**Figure 5:**
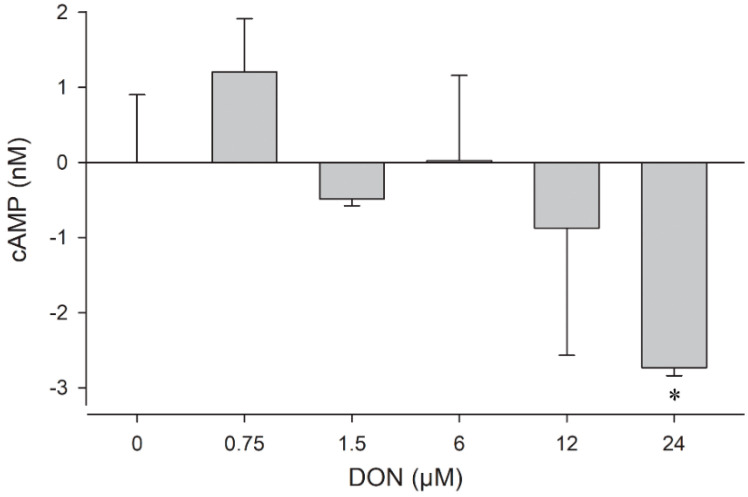
Dose-dependent changes in the cAMP level in murine astrocytes after incubation for 1 h. The data are representative of two independent experiments and are expressed as mean ± SD of three parallel incubations. Significant differences (*p* < 0.05) determined by Student’s t-test are indicated with asterisks (*).

In the present study, we have, therefore, investigated if exposure to DON could result in changes in the cAMP level in mouse glial cells. The assay revealed that the intracellular cAMP concentrations were affected in astrocytes already after incubation for 1 h (Figure 5), whereas no change was observable in the microglia after this time (data not shown). In contrast, 4 µM of the positive control substance forskolin, an activator of adenylate cyclase, raised cAMP to about 23 nM in astrocytes and 2 nM in microglia already after 15 min (data not shown). The impact of DON on the astrocytes was dose-dependent, resulting in a slight increase to +1 nM at sub-µmolar level, but a substantial decrease at concentrations above 6 µM, with −3 nM at 24 µM DON.

To our knowledge, this is the first time that DON-induced changes in the cAMP level of astrocytes are reported. This mode of action might contribute to understand DON neurotoxicity since cAMP activation has an upstream position in pathways connected to stress alleviation or cell death [60]. Since we found that the cAMP levels were increased by exposure to 0.75 µM DON and dropped below their physiological level at 6 µM DON, it could be possible that the first mechanisms to repair the toxin-mediated stress had been initiated, while higher DON levels suppressed the generation of cAMP, switching the outcome to cell death.

### 2.5. DON Impact on Glial Cells in Primary Hippocampal Cultures

**Figure 6:**
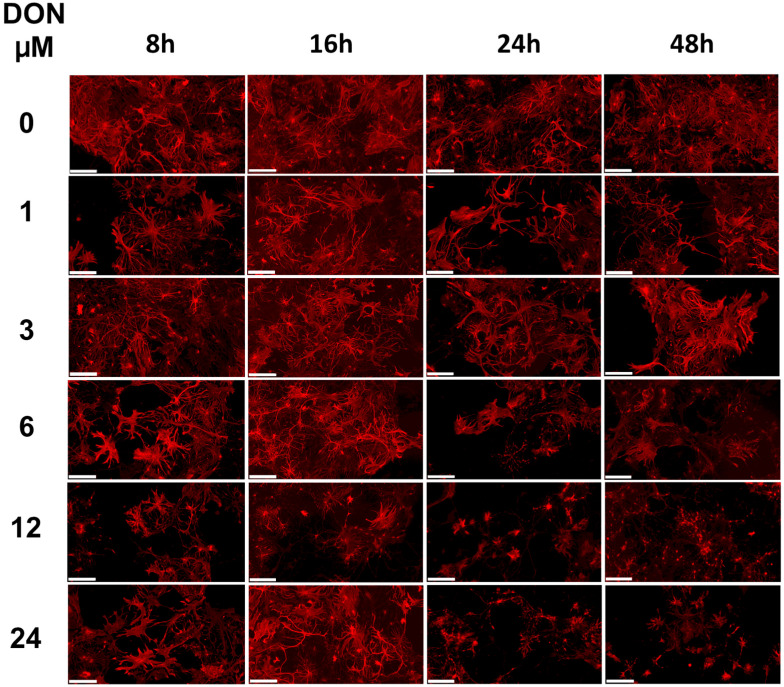
Impact of DON exposure on glial cells in primary hippocampal cultures. Three independent cultures were prepared and analysed for changes in the astrocyte morphology using GFAP staining. Representative images upon treatment with 0, 1, 3, 6, 12, or 24 µM DON for 8, 16, 24, or 48 h. Scale bars: 100 µm.

Cultured primary hippocampal cells, containing astrocytes, microglia, and neurons are a useful model to investigate neuronal vulnerability to toxicants [61]. After their preparation from mouse pup brains, we incubated the hippocampal cells for 15 days, allowing them to interconnect and to fully develop neuronal structures. Exposure to increasing DON levels had a concentration- and time-dependent effect on the astrocyte morphology that became visible by staining of the glial fibrillary acid proteins (GFAP) (Figure 6). Changes could be observed for 6 μM DON after 24 h exposure but were already detectable after 8 h exposure at higher DON concentrations. 

The intermediate filament (type III) GFAP is a fundamental component in the cytoskeleton of astrocytes, shaping cell architecture, providing mechanical strength, regulating filament assembly during mitosis, and helping to support adjacent neurons [62]. There is also evidence for a role in glutamate transporter trafficking and membrane anchoring in astrocytes. The protein is post-translationally modified by phosphorylation at numerous sites, which increases during mitosis. Any disturbance of this process might affect the cell cycle progression in astrocytes, comparably for example, to the changes found in our test using an astrocytes cell culture after 24 h exposure to 5 and 10 µM DON (Figure 3). In these cultures, the reduction of the astrocytic metabolism was almost maximal for DON at 5 µM (Figure 2a) and a negative impact on the astrocytic cAMP concentrations was detected at doses higher than 6 µM (Figure 5). 

Phosphorylating enzymes such as protein kinase A are often cAMP-dependent and hence influenced by intracellular cAMP-levels. The observed impact of DON at concentration higher than 5 to 6 µM in the astrocytes may subsequently affect the GFAP distribution, which is congruent with the morphological changes induced in primary hippocampal cultures by the same DON concentration (Figure 6). Similarly, when primary rat hippocampal cultures were exposed to DON for 48 h and stained for GFAP in a previous study [35], morphological changes in astrocytes were observable above 10 µM. Interestingly, changes in the morphology of gliocytes and myenteric neurons in the jejunum had been observed in vivo in rats fed with 0.75 mg/kg feed [24].

Altogether, our data illustrated the impact of µmolar DON concentrations on the physiology of astrocytes, both in vitro in a monoculture cell line and ex vivo in primary hippocampal co-cultures.

### 2.6. DON Impact on Neurons in Primary Hippocampal Cultures

**Figure 7:**
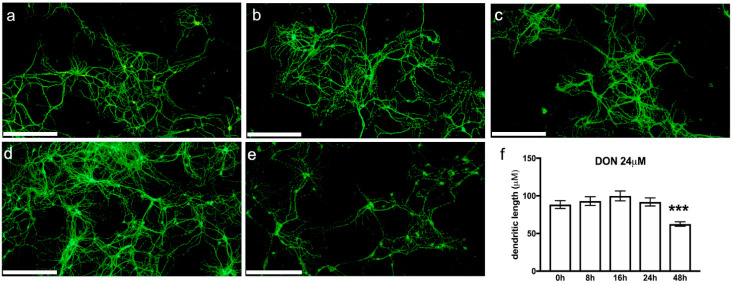
Representative images of murine primary hippocampal cultures: (**a**) untreated, (**b**) treated with 24 µM DON for 8 h, (**c**) 16 h, (**d**) 24 h, or (**e**) 48 h. Neurons were identified using MAP2 labelling. Scale bars: 250 µm. (**f**) Quantification of the dendritic lengths. Data are presented as mean ± SEM, *n* =40–80, *** significant difference (*p* < 0.001) as compared to the other time points.

Next, we examined changes in the neuron morphology in our mouse primary hippocampal cultures using fluorescence immunostaining of the microtubule-associated protein 2 (MAP2) (Figure 7). The integrity of the neuronal shape is of critical importance for nerve cell functionality, conditioning signal transmission, and synaptic connectivity [63]. Several pathologies, particularly neurodegenerative diseases, are characterised by neuronal loss and by a reduction of the dendritic arborisation [64,65].

The cytoskeleton is an important endogenous determinant of neuronal morphology. Involved in providing stability to the cytoarchitecture of neurons is MAP2, an abundantly occurring, high-molecular weight polypeptide. MAP2 is essential for tubulin polymerisation into microtubules and influences dendritic lengths. Thus, it is an established marker for the somato-dendritic compartment of nerve cells.

We examined the impact of 0, 1, 3, 6, 12, or 24 µM DON exposure for 8, 16, 24 or 48 h on the neuronal shape (Figure 7a–e) and observed that the dendritic lengths were significantly shortened after 48 h exposure to 24 µM DON (Figure 7f). This result gives further evidence of DON’s neurotoxic potential. It confirms the impact of chronic low-dose DON exposure, which we previously had detected in neurons of DON-treated mice by using c-fos expression [10].

## 3. Conclusions

The mycotoxin DON has demonstrated a neurotoxic potential in different animal studies. In the present project, we have investigated the underlying mechanisms of action in mouse astrocytes, microglia, and primary hippocampal cultures. We determined that low µmolar DON concentrations reduced the viability of astrocytes, affected their cell cycle progression, and decreased cAMP levels. In microglia, DON induced cytotoxicity and led to an increased TNF-α secretion. DON exposure affected the morphology of primary neurons by reducing the dendritic arborisation. Taken together, these findings showed that DON interfered with the cellular metabolism/physiology of glial and neuronal cells, which could explain some of the observed neurotoxic in vivo effects.

## 4. Materials and Methods

### 4.1. Chemicals

Deoxynivalenol (DON) (D-0156) was obtained from Sigma-Aldrich (St. Louis, MO, USA). It was dissolved in water for use in the cell exposure studies. Dimethyl sulfoxide (DMSO) and forskolin were from Sigma-Aldrich. Alamar blue, propidium iodide, RNAse A, and DRAQ5 were purchased from ThermoFisher Scientific (Waltham, MA, USA). Dulbecco’s Modified Eagle Medium (DMEM), phosphate buffered saline (PBS), trypsin-versene (EDTA) mixture, penicillin-streptomycin mixture, and foetal bovine serum EU standard (FBS) were from Lonza (Verviers, Belgium). The CellTox Green cytotoxicity assay and cAMP-Glo assay were bought from Promega (Madison, WI, USA). BD BioCoat Cellware poly-L-lysine 12 mm coverslips and the TNF-α ELISA were obtained from BD Biosciences (Bedford, MA, USA). The IL-1β ELISA was from R &D Systems (Minneapolis, MN, USA).

### 4.2. Cell Culture Conditions

Murine C8-B4 microglia cells (ATCC CRL-2540; monocytes from cerebellum of C57BL/6 mice (*Mus musculus*) and C8-D1A astrocytes (ATCC CRL-2541; astrocyte type I clone from C57BL/6 mouse cerebellum) were obtained from American Type Culture Collection (ATCC, Manassas, VA, USA) and grown in DMEM-supplemented with 10% FBS and 1% penicillin/streptomycin. The microglia and astrocytes were cultured at 37 °C under 5% CO_2_ in a humidified incubator (NuAire, Plymouth, MN, USA) and sub-cultivated by trypsination once/twice per week in a 1:5/1:10 split ratio, respectively. They were allowed to grow as monolayers in 75 cm^2^ cell culture flasks with filter screwcaps (Techno Plastic Products, Trasadingen, Switzerland) until 80–100% confluence was reached. The microglia and astrocytes were plated at 90,000/cm^2^ and 45,000/cm^2^, respectively, 24 h prior to the test, which resulted in approximately 90% confluence at the day of exposure as observed by light microscopy (Leica DMIL, Solms, Germany). The cell culture medium was refreshed before performing the exposure test.

### 4.3. Metabolic Activity

Metabolic activity of astrocytes and microglia was measured after exposure to 0.8, 1.6, 3.1, 6.3, 12.5, and 25 µM DON for 24 h using the Alamar blue assay according to the manufacturer’s protocol (ThermoFisher Scientific). The dark blue oxidised form of Alamar Blue (resazurin) is reduced to a highly fluorescent form (resorufin) by mitochondrial or cytoplasmatic enzymes. The fluorescence (Ex: 555 nm, Em: 585 nm) was quantified using the Spectramax i3x plate reader (Molecular Devices, San Jose, CA, USA). The measured fluorescence intensity is proportional to the number of viable cells and a measurement of the metabolic fitness of the cells [40].

### 4.4. Cytotoxicity

Cytotoxic effects of DON (0.8, 1.6, 3.1, 6.3, 12.5, and 25 µM) in astrocytes and microglia were analysed after 24 h exposure with CellTox^TM^ Green Dye (1:2000). The dye was added to the cells as described by the manufacturer, and fluorescence signals (Em: 485 nm/Em: 520 nm) were quantified with a Spectramax i3x plate reader using the well scan function by reading 32 different points/well in all wells. The cytotoxicity data were normalised by the total cell numbers, which were determined by staining with the nuclear stain DRAQ5 (1:500, 30 min) and counting with the Spectramax i3x plate reader equipped with a microscopic module (MiniMax300imaging cytometer, Molecular Devices), Ex: 625 nm, Em: 713 nm.

### 4.5. Expression of Cytokines

TNF-α secretion was measured in the supernatants of astrocytes and microglia exposed to 0.38, 0.75, 1.5, 3 and 6 µM DON for 24 h. After centrifugation (500× *g*, 10 min) to remove cell debris, TNF-α was quantified by enzyme-linked immunosorbent (ELISA) as instructed by the manufacturer using the Spectramax i3x plate reader. IL-1β levels in the cell supernatants were measured by ELISA following the manufacturer’s guidelines. Additionally, IL-1β was measured in microglia cells that had been pre-incubated with 0.05 or 0.1 ng/mL LPS for 3 h and exposed to 0.75 or 1.5 µM DON for 24 h.

### 4.6. Cell Cycle Analysis

For cell cycle analysis, astrocytes and microglia were exposed to 1, 2.5, 5, and 10 µM DON for 24 h, washed with PBS and fixed with ice-cold 70% ethanol overnight at −20 °C. The cells were then washed with PBS, incubated with propidium iodide (10 µg/mL)/RNase A (100 µg/mL) in PBS for 30 min at 37 °C before analysis by flow cytometry (Accuri C6; BD Biosciences). Single cells were gated and a minimum of 10,000 cells acquired and analysed. Data acquired were analysed by Accuri CFlow Plus software (version 1.0.227.4; BD Biosciences).

### 4.7. Determination of cAMP Levels

Astrocytes and microglia were exposed to 0.75, 1.5, 6, 12 and 24 µM DON for 1 h, and intracellular cAMP concentrations were measured using the cAMP-Glo assay according to the manufacturer‘s guidelines (Promega) using the Spectramax i3x plate reader. The cAMP agonist forskolin (4 µM; 15 min exposure) was used as positive control.

### 4.8. Primary Hippocampal Cultures from Mouse Pups

Primary hippocampal cell cultures were prepared as previously described [66]. Briefly, P0-P3 mice pups from C57BL/6J:129SvPas (50:50) mice were decapitated. Hippocampi were dissected and digested with papain (20 U/mL, Worthington Biochemical Corp., Lakewood, NJ, USA). The cells were plated (8–10 × 10^4^ cells/well) on poly-L-lysine-coated coverslips in 24-well plates. Cultures were maintained for 15 days in vitro (DIV) with half of the medium (Neurobasal A medium: 2 mM glutamax, 0.5 mM glutamine, penicillin/streptomycin, supplemented with 2% B27) (GIBCO, Loughborough, UK) changed every 5 to 7 days. The cells were incubated for 8 h, 16 h, 24 h, or 48 h with 0, 1, 3, 6, 12, or 24 μM DON, washed in cold 0.1 M, pH 7.4 PBS and fixed with 4% paraformaldehyde (32% diluted to 4% before use; ThermoFisher Scientific). The coverslips with fixated cells were washed three times with cold PBS and kept at 4 °C until processing.

### 4.9. Determination of Neural and Glial Markers by Immunofluorescence

Primary hippocampal cultures were incubated in PBST blocking solution (PBS with 0.2% Tween 20 and 5% normal goat serum; Sigma-Aldrich) for one hour at room temperature (RT) (20–22 °C), and then overnight at 4 °C in blocking solution with mouse anti-microtubule-associated protein 2 (MAP2, (1:1000; Sigma-Aldrich) and rabbit anti-glial fibrillate acid protein (GFAP, 1:5000; DakoCytomation, Glostrup, Denmark) primary antibodies. The cells were washed three times in PBST and incubated for 2 h at RT with goat anti-mouse antibodies coupled to AlexaFluor 647 (1:500; A21240, Molecular Probes; Eugene, OR, USA) and goat anti-rabbit antibodies coupled to AlexaFluor 594 (1:2000; A11012, Molecular Probes). After three washes in PBST and incubation in PBST for 2 h at RT, nuclei were stained with 4′,6-diamidino-2-phenylindole dihydrochloride (DAPI); ThermoFisher Scientific) (1 µg/mL in PBS) for 5 min in the dark. After the removal of the staining solution, the cells were washed in PBS, mounted with ProLong™ Gold Antifade mounting medium (Molecular Probes) and kept at −20° protected from light until imaging. Images were acquired using the slide scanner NanoZoomer 2 HT with fluorescence module L11600-21 (Hamamatsu Photonics, Hamamatsu City, Japan). Single RGB colour acquisition was made in the epifluorescence mode with the time delay integration 3-chip camera equipped with a filter-set optimised for DAPI, tetramethylrhodamine and cyanine-5 detection. The acquisition was performed using a dry 20x objective (NA: 0.75). The 40x resolution was achieved with a lens converter. The latter mode used the full capacity of the camera (resolution: 0.23 µm/pixel).

### 4.10. Image Analysis

Dendritic lengths were measured on images of neurons labelled with MAP2 (randomly selected from three independent cultures) using the NeuronJ plugin from the Image J package Fiji (Available online: https://imagej.net/software/fiji/ (accessed on 13 December 2021), which allows semiautomatic tracing and measurement of neurites [67]. 

### 4.11. Statistical Analysis

Data of the cellular assays were analysed by using Sigma Plot version 12.0 (Alfasoft AS, Lillestrøm, Norway). Statistical significance (*p* < 0.05) was assessed using 1-way-ANOVA, followed by the Holm-Sidak post-test. When data failed the equal variance test, ANOVA could not be used. Instead, the Student’s t-test was performed for comparing the control group to the treated group. Standard error of the mean (SEM) was used for results of the cell line assays, where the mean of several independent experiments with three or more replicates were shown. Standard derivation (SD) was used when one representative experiment with three or more replicates was shown. Statistical analysis of dendritic lengths was performed with Graph-Pad Prism V7 (Available online: https://www.graphpad.com/ (accessed on 13 December 2021) using the non-parametric Kruskall-Wallis test followed by Dunn’s post hoc comparison.

## Data Availability

The data sets generated during the current study are available from the authors upon request.
